# Transcriptome Analysis of *Amyloodinium ocellatum* Tomonts Revealed Basic Information on the Major Potential Virulence Factors

**DOI:** 10.3390/genes11111252

**Published:** 2020-10-24

**Authors:** Omkar Byadgi, Fabio Marroni, Ron Dirks, Michela Massimo, Donatella Volpatti, Marco Galeotti, Paola Beraldo

**Affiliations:** 1 Section of Animal and Veterinary Sciences, Department of Agricultural, Food, Environmental and Animal Sciences (DI4A), University of Udine, 33100 Udine, Italy; michela.massimo@uniud.it (M.M.); donatella.volpatti@uniud.it (D.V.); marco.galeotti@uniud.it (M.G.); paola.beraldo@uniud.it (P.B.); 2 Department of Agricultural, Food, Environmental and Animal Sciences, University of Udine, Via delle Scienze 206, 33100 Udine, Italy; fabio.marroni@uniud.it; 3 IGA Technology Services, Via Jacopo Linussio, 51, 33100 Udine, Italy; 4 Future Genomics Technologies B.V, 2333 Leiden, The Netherlands; dirks@futuregenomics.tech

**Keywords:** ectoparasite, *Amyloodinium ocellatum*, tomonts, transcriptome, virulence proteins

## Abstract

The ectoparasite protozoan *Amyloodinium ocellatum* (AO) is the etiological agent of amyloodiniosis in European seabass (*Dicentrarchus labrax*) (ESB). There is a lack of information about basic molecular data on AO biology and its interaction with the host. Therefore, de novo transcriptome sequencing of AO tomonts was performed. AO trophonts were detached from infested ESB gills, and quickly becoming early tomonts were purified by Percoll^®^ density gradient. Tomont total RNA was processed and quality was assessed immediately. cDNA libraries were constructed using TruSeq^®^ Stranded mRNA kit and sequenced using Illumina sequencer. CLC assembly was used to generate the Transcriptome assembly of AO tomonts. Out of 48,188 contigs, 56.12% belong to dinophyceae wherein *Symbiodinium microadriaticum* had 94.61% similarity among dinophyceae. Functional annotations of contigs indicated that 12,677 had associated GO term, 9005 with KEGG term. The contigs belonging to dinophyceae resulted in the detection of several peptidases. A BLAST search for known virulent factors from the virulence database resulted in hits to Rab proteins, AP120, Ribosomal phosphoprotein, Heat-shock protein70, Casein kinases, Plasmepsin IV, and Brucipain. Hsp70 and casein kinase II alpha were characterized in-silico. Altogether, these results provide a reference database in understanding AO molecular biology, aiding to the development of novel diagnostics and future vaccines.

## 1. Introduction

The ectoparasite protozoan *Amyloodinium ocellatum* (AO) is the etiological agent of amyloodiniosis, of which the European sea bass (*Dicentrarchus labrax*) is one of the many susceptible species. The lifecycle of *Amyloodinium ocellatum* is direct and triphasic and can be completed in less than a week provided the environmental conditions are favorable (optimal temperature range 24–28 °C). The trophont is the parasitic stage feeding directly from the host on gill and skin epithelia, to which it adheres by rizhoids [1,2]. Two to six days after feeding, the trophont detaches from the host and encysts on inert substrates transforming into the tomont, free-living cystic reproductive stage from which it can hatch up to 256 dinospores by asexual reproduction (the number of dinospores produced per tomont is proportional to the trophont size) [3,4]. The dinospore is the free-swimming infective stage and after its adhesion to a new host, it transforms into a trophont in a few minutes.

Although major losses incurred by amyloodiniosis are recorded during ESB grow out, there are currently no licensed and effective measures to control the infestation. However, during natural outbreaks in ESB, the expression of host-related immune genes has been investigated [5]. At present, there is no practical solution to culture AO continuously in-vitro in large-scale industrial operations and using fish as a host to maintain the parasite is time-consuming and involves fish sacrifice. Moreover, the scarcity of molecular data has hindered the progression of our understanding of molecular biology, especially concerning the mechanisms contributing to the virulence of the parasite. Furthermore, the lack of a whole-genome sequence of AO considerably limits any comparative “omics” analysis (transcriptome, proteome). Therefore, in order to broaden the repertoire of molecular data, a de novo Transcriptome sequencing study of AO tomonts was performed. This approach to procuring information on the transcriptome of a parasite can help to discover some important genes and understand their molecular process during parasite development, reproduction, and host interactions, as well as identifying potential vaccine candidates and drug targets [6]. There are few studies related to fish parasites transcriptome. It was observed that the most abundant transcript in *Ichthyophthirius multifiliis* trophonts was stage-specific and most of the sequence was related to metabolic activates [7]. In *Cryptocaryon irritans,* it was recorded that most of the differentially enriched genes in tomonts are involved in cell division mainly to ensure parasite continuity [6]. Interestingly, under a temperature of 12 °C, tomonts of *C. irritans* indicated inhibition of genes correlated with normal cell development but showed an upregulation of genes related to parasite survival and cell entry [8].

Hence, in the present study, Illumina sequencing was used to generate the transcriptome assembly of AO early tomonts in order to create the reference database. We would like to emphasize that, immediately after their detachment from the fish, trophonts (feeding parasitic stage) become tomonts (free-living cystic reproductive stage), making their use inevitable for this study. Furthermore to obtain the pure trophonts without host tissue contamination for sequencing is hardly achievable. The data were used to determine several functional classifications of the sequence from tomonts to clarify the biological functions of harmful AO in ESB. The objectives of this study are: (1) to generate de novo transcriptome sequence and assembly of *Amyloodinium ocellatum* tomonts, (2) to determine the functional classification of assembled transcripts into cellular components and the biological process, and (3) to identify several potential virulence proteins based on blast similarity with AO contigs and to characterize heat shock proteins 70 and casein kinase II alpha from AO transcriptome data.

## 2. Materials and Methods

### 2.1. Parasite and Total RNA Preparation for Sequencing

Originally AO trophonts were collected during a natural infestation in European sea bass (ESB, *Dicentrarchus labrax*) farmed in the delta area of the Po River (Italy). Subsequently, the parasite was preserved as tomont in a in-vitro hibernation state (at 17 ± 0.5 °C) and regularly in-vivo reactivated to produce new parasite generation for research purposes or for the renewal of the AO hibernated stocks. Therefore, for this study, AO early tomonts were only resulting from experimental ESB infestation, managed in a 300 L aquarium with water temperature 24–26 °C and salinity 28‰, and collected 5–6 days after infection. Parasite collection and preservation were performed by following the standard operating protocol as described [9]. In synthesis, a rapid detachment of trophonts from ESB-infested gills and skin epithelia was induced by a freshwater bath in a jar; after 1–2 min fish were removed and 40‰ sterile seawater was added to reach the 20‰ salinity. The content was poured (100 μm nylon filter mesh) and then, after 10 min, early tomonts were collected, purified by Percoll^®^ gradient, washed several times in sterile seawater, concentrated by centrifugation and immediately used for RNA extraction. A negligible amount of host cells could be present in the early tomonts pellet.

Total RNA was extracted using Qiagen miRNeasy Mini kit according to the manufacturer’s instructions (Qiagen GmbH, Hilden, Germany). The RNA quality was confirmed using the ratios A260/280 and A260/230 (Thermo fisher scientific Inc., Waltham, MA, USA). RNA concentration and integrity were analyzed with a Bioanalyzer 2100 total RNA Nano series II chip (Agilent, Santa Clara, CA, USA). Total RNA from tomonts was shipped using dry ice to Future Genomics Technologies, The Netherlands for Illumina sequencing.

Ethics Statement: All the experiments included in the present study have been carried out in the facilities (fish stabularium ID 5E7A0) of Department of Agricultural, Food, Environmental and Animal Sciences (University of Udine), as authorized by the Italian Ministry of Health (decree n 14/2018-UT, 12/11/2018). The animal care and protocols adopted adhere to the Directive 2010/63/EU of the European Parliament, implemented at a national level by the D.L. n. 26 of 4 March 2014.

### 2.2. Sequence Assembly

cDNA libraries were made using the following kit, TruSeq^®^ Stranded mRNA Sample Preparation manuals (Illumina, San Diego, CA, USA). The libraries were sequenced using Illumina HiSeq™ 2500 instrument (Illumina, San Diego, CA, USA) with paired-end 2 × 100 nucleotide multiplex, according to the manufacturer’s instructions. Bbmap was used to remove the remaining Illumina adapters from the sequencing reads. Bowtie2 (very sensitive settings) was used to align the cleaned reads against the *Dicentrarchus labrax* transcriptome CDS (diclab1_cds.fasta; downloaded from NCBI) and unaligned reads were recovered in FASTQ format. CLC assembly cell 4.4.1 was used for de novo contigs assembly of the unaligned reads; several kmer and bubble size combinations were used to optimize the statistics of the assembly (N50 and number of contigs). Diamond BLAST was used to compare assembly_k30_b200 with the NCBI-nr database at an *E*-value cutoff of 1 × 10^−5^; only the best hit was reported. The BLAST results indicated that a small subset of contigs corresponded to plant sequences (Taxon ID 33090; Viridiplantae; 3924 contigs), probably because of aerosol contamination during the Illumina library prep; these contigs were removed.

### 2.3. Assigning Functional Annotations to Transcripts Belonging to Alveolata

Transcripts assigned to Alveolata were selected for functional annotation. Assembled transcripts were blasted against the Swissprot database [10] using BLASTx [11]. Results with an *E*-value of 10^−5^ or lower were retained. The resulting Swissprot ID to which the transcripts mapped was used to lookup annotations corresponded to the transcript. GO terms were searched using a text file made freely available by the European Bioinformatics Institute (EBI) as part of the GOA project (ftp://ftp.ebi.ac.uk/pub/databases/GO/goa/UNIPROT/goa_uniprot_all.gaf.gz); GO terms are reported separately by GO category, i.e., Biological Process (BP), Cellular Components (CC), Molecular Function (MF). KEGG classification was obtained by searching the SwissProt ID in the KEGG database using KEGGREST [12]. eggNOG classification [13] was also obtained. eggNOG terms are returned as associated with Ensembl terms. The Uniprot web mapping tool (https://www.uniprot.org/uploadlists/) was used to convert the Uniprot ID of the alveolata transcripts to Ensembl ID. Ensembl ID was then searched in the eggNOG database and the relative annotations (if any are returned).

### 2.4. Assigning Taxonomy to Transcripts

A table summarizing the taxonomy of the assembled transcripts was provided. For each transcript, the assigned species is indicated. Using the tool (https://github.com/zyxue/ncbitax2lin) we assigned taxonomy to each transcript. The taxonomy includes all the “official” taxonomy levels, plus several unofficial levels that are commonly used in studies. For example—we also returned the classification to “alveolata”, under the informal taxonomy header “no rank 1”.

### 2.5. Identification of Peptidases and Virulence Factors

All contigs were compared to protein sequences in the MEROPS “pepunit.lib” database [14] to identify putative proteases. A BLASTx was performed with *E*-value ≤ 1 × 10^−3^. To access a larger data pool of known virulent factors, a custom BLAST database was generated consisting of sequences of all virulent factors included in the virulence protein databases MvirDB [15] and ProtVirDB [16].

### 2.6. In Silico Analysis of AO Heat Shock Protein 70 and Casein Kinase II Alpha

From the output of the virulence protein database of AO tomonts, the hsp70 and casein kinase II alpha sequence was searched. The retrieved sequence was subjected to BLASTx using default settings from NCBI. Subsequently, the confirmed sequence was run in The ExPASy proteomic tool (available online: http://expasy.org/tools/) to predict molecular weight (kDa), pI of the protein sequence. SMART software (available online: http://smart.embl-heidelberg.de/) was used to determine protein domains. The protein prediction PSIPRED server [17] was used to determine the secondary structure and phyre2 server for 3D structure [18].

## 3. Results

### 3.1. De Novo Sequence Assembly of AO Transcriptome

The raw FastQC file was deposited in the National Center for Biotechnology Information (NCBI) Sequence Read Archive (SRA) database under the accession number SRX5567002 and Bio project accession number PRJNA528860. 48,188 of the cleaned contigs had a BLASTx hit against the nr database (Table 1). The set of contigs that had an alveolata as the best hit was extracted and included in the FASTA file and the length of the contigs was determined (Supplementary Figure S1). Out of 48,188 contigs, 93.4% belong to Eukaryota and 56.12% to dinophyceae. The majority of the contigs had a 94.61% similarity with the species *Symbiodinium microadriaticum* (Table 2).

### 3.2. Functional Annotation of AO Contigs

**Figure 1:**
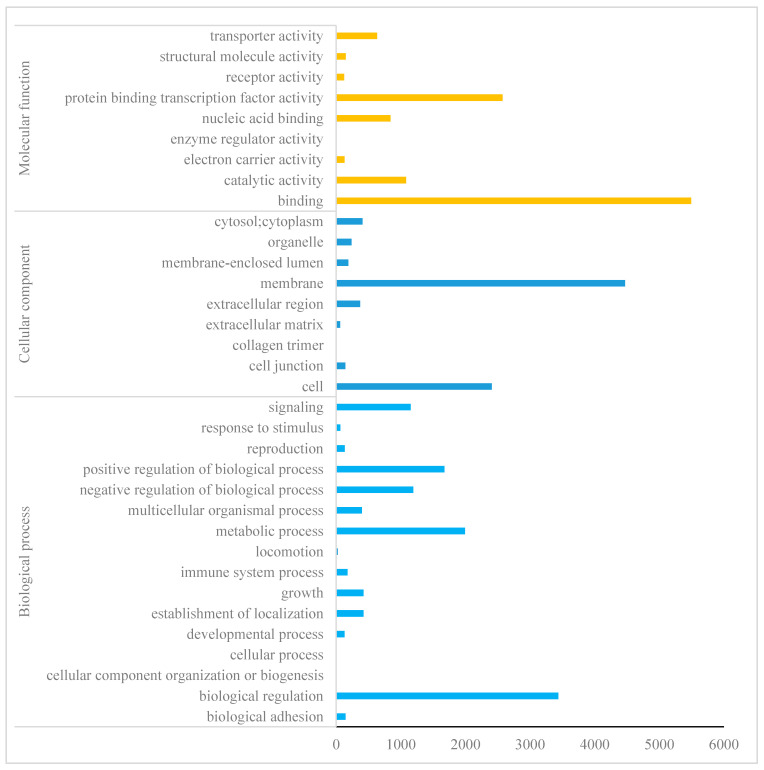
Gene ontology (GO) enrichment analysis of *Amyloodinium ocellatum*. The results of GO enrichment analysis are classified into three categories: biological process, cellular component and molecular function. The *y*-axis indicates the gene functional classification of GO, the *x*-axis indicates the corresponding number of genes.

**Figure 2:**
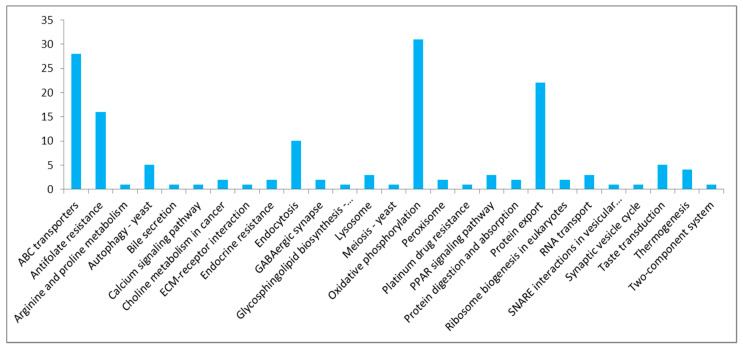
KEGG enrichment analysis of *Amyloodinium ocellatum* tomonts.

In total, 13,667 transcripts out of 30,083 had a BLASTx hit against Uniprot; 12,677 had at least an associated GO term, 9005 had at least an associated KEGG term, and 2953 had at least an associated eggNOG term. The AO tomont genes were mainly annotated into biological processes, cellular components and molecular functions (Figure 1). The most annotated are molecular functions, wherein the highest number of enriched genes was related to binding. Among the cellular component, membrane and cell molecules were found to be enriched. Most of the genes related to biological regulation and metabolic processes were found to be highly enriched from the biological process. KEGG analysis indicated the highest number of ABC transporters, antifolate resistance, oxidative phosphorylation and protein export (Figure 2).

### 3.3. Peptidases Identified from AO Transcriptome Data

*Amyloodinium ocellatum* expresses a wide range of peptidases (Table 3) as reflected in the results retrieved from the BLASTx search against MEROPS pepunit.lib database (Rawlings et al., 2006). The search resulted in the detection of several peptidases with contigs of AO (*E*-value ≤ 1 × 10^−5^); excluding hits to non-peptidase, homologues and inhibitors (Table 3). The description of all these peptidases among the peptidase family is under progress to determine the major group and dominant peptidase enzyme in AO.

### 3.4. Potential Virulent Factors from AO Transcriptome

In addition to data mining for already described virulence factors in related protozoa, a BLAST search using a custom made virulence database, consisting of the MvirDB [15] and ProtVirDB [16] databases, was performed. The search resulted in several contigs similar to potential virulence factors belonging to Adhesin, invasion, establishment and proteases (Table 4). Hits matching to Rab proteins, Rab11B, RabA and Rab 7A were the most abundant. Therefore, all these major proteins identified could be considered as potential virulence determinants in AO.

### 3.5. Sequence Analysis of AO Heat Shock Protein 70 and Casein Kinase II Alpha

**Figure 3:**
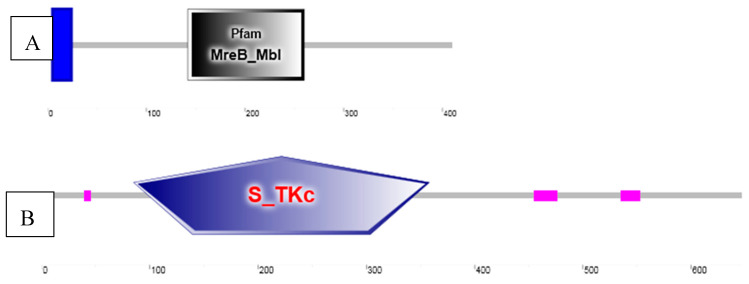
Heat shock protein 70 (**A**; Contig_50520) and casein kinase II alpha (**B**; Contig_10331) domains in AO tomonts. Domain structure predicted by SMART analysis of the amino acid sequence from AO transcriptome database.

AO heat shock 70 protein has 410 amino acids with Theoretical pI of 5.76 and a molecular weight of 43.8 kDa (Supplementary Figure S2). Casein kinase II alpha has 647 amino acids with a theoretical pI of 5.37 and a molecular weight of 70.5 kDa. The transmembrane region starting from five amino acids was predicted in hsp70 and a Pfam: MreB_Mbl domain was recorded from 142 amino acids with an *E*-value of 9.2 × 10^−10^ (Figure 3A). The protein kinase domain S_TKc was predicted in the amino acid sequence of AO casein kinase (Figure 3B). The predicted secondary structure of the hsp70 (Supplementary Figure S2a) and casein kinase (Supplementary Figure S2b) was found to contain one helix. 

## 4. Discussion

In Mediterranean aquaculture, amyloodiniosis can lead to serious economic impacts on ESB farmed inland or in lagoon-based semi-intensive and intensive systems. It can cause dramatic economic losses, primarily during the warmer months, with acute mortality around 100%, depending on farming conditions and parasite burden.

To the best of our knowledge, there are no studies on the transcript profile of *Amyloodinium ocellatum*. However, few reports are related to host immune response throughout AO infestation. As reported in previous studies, Interleukin-1 (IL-1) and Tumor Necrosis Factor-α (tnf-α) were activated in infested ESB reared in an aquaponics system [19]. Natural AO outbreaks in ESB resulted in pronounced and sustained inflammation (*il-8, cc1,* and *cox-2* upregulation) involving many novel molecules (Hepcidin) at the site of AO attachment to the gills, as demonstrated by a study highlighting the immediate local immune responses of ESB to AO infestation [5]. Still, the paucity of molecular data on the parasite itself hindered the progression of our understanding of the mechanisms contributing to the parasite virulence. Furthermore, the lack of a whole-genome sequence of AO considerably limits any comparative “omics” analysis (Transcriptome, Proteome). Against this background, in order to obtain a reference database of AO tomonts, all the raw reads were first de novo assembled using the tomont stage.

At present, the in vitro culture of AO without a host is reliable by using the G1B cell line (ATCC^®^ CRL-2536TM); however, this host-free system is complex and does not allow us to obtain a large quantity of parasite. Alternatively, “early-tomontised” trophonts are easy to obtain in large quantity by means of an AO controlled infestation (even without necessarily sacrificing the fish) and with negligible bacterial or host contamination. Still, in this study, an alignment strategy aimed at removing these contaminations was applied using ESB transcripts and mapping to the bacterial database as reference. In fact, some dinoflagellates, among them *A. ocellatum*, are known as organisms able to perform “phagotrophy” in order to get nutrients from the host cell cytoplasm, as observed ultra-structurally [1]. Therefore, the remaining unaligned sequence was considered as belonging to AO tomonts, later de novo assembled and used for further analysis on a comprehensive functional gene annotation.

Overall, in the group of Dinophyceae, there are only three genome data available from three species, *Symbiodinium minutum* (603.73 Mb), *Symbiodinium microadriaticum* (808.23 Mb), *Prorocentrum minimum* (29.35 Mb). Even though the group of dinophyceae consists of several free planktonic and bloom causing organisms, there are few difficulties to sequence the genome of dinoflagellates being their size extremely large and irregular. This genome is expensive to sequence (difficult to predict the size of AO, unless we conduct the complete sequence), it contains a high number of methylated nucleotides and displays unusual regulatory mechanism [20]. The unusual genome structure of dinoflagellates raises the question of whether gene regulation in dinoflagellates is controlled by transcription factors. However, both transcriptomic and genomic analyses have shown there is a paucity of sequence-specific transcription factors in dinoflagellates, consistent with a constant, steady transcription of most genes with fewer genes under sequence-specific transcriptional control [21].

The Gene Ontology (GO) provides a system for hierarchically classifying genes or gene products into terms organized in a graph structure (or an ontology) [22]. The terms are grouped into three categories: molecular function (describing the molecular activity of a gene), biological process (describing the larger cellular or physiological role carried out by the gene, coordinated with other genes) and cellular component (describing the location in the cell where the gene product executes its function). Each gene can be described (annotated) with multiple terms. In our study, AO transcriptome indicated the highest number of genes related to biological regulation and metabolic activity from biological processes. This was expected, as the tomont is the dividing stage, which involves the cell cycle as well as the generation and use of energy for metabolic activities [23]. GO annotation under molecular function was mostly enriched with genes related to binding. It is noteworthy that the GO indicated binding molecules as AO is an ectoparasite that adheres to ESB gills during infestation and therefore the molecules related to cell adhesion, stress response, circadian rhythm, dormancy process, and cell communication, are crucial. A previous study on *C. irritans* tomont transcriptome indicated that proteins, such as the external encapsulating structure, cell projection proteins and proteinaceous extracellular matrix, were annotated under the GO category cellular component and the authors emphasized that the proteins related to cellular component could be considered as potential diagnostic markers of the tomont stage [5]. In a study on *Aurantiochytrium* sp. under cold stress, “cellular process”, “binding” and “metabolic process” were also the largest proportion, and fatty acid biosynthetic processes were also influenced by cold stress [24]. The results above revealed the importance of biological processes and molecular function of the protozoans submitted to a low-temperature regime. Altogether, the GO annotated data from AO provide a basis to identify important molecules, which are necessary for AO survival during the infestation and are involved in amyloodiniosis pathogenesis. Further studies should be directed to understand the DEG and GO annotation differences among different life stages of AO.

Our results on KEGG analysis from AO contigs indicated a high number of ABC transporters, Antifolate resistance, oxidative phosphorylation and protein export. The ATP-binding cassette (ABC) transporters couple ATP hydrolysis to active transport a wide variety of substrates such as ions, sugars, lipids, peptides, proteins, and drugs [8]. The protein export is the active transport of proteins from the cytoplasm to the exterior of the cell. In *C. irritans* submitted to the KEGG analysis, various genes that are involved in the metabolism, transporters and endocytosis were also identified [6].

Peptidases are proteolytic enzymes that have been suggested as drug targets for parasitic diseases [25]. In parasites, peptidases are involved in host–parasite interactions, destruction of host tissues, parasite migration, and acquisition of essential nutrients for survival, growth, development and proliferation required throughout infestation. Therefore, we used the *MEROPS* database to verify if our AO contigs contain peptidase sequences. MEROPS contains information on about more than 4000 peptidases and nearly 700 inhibitors. We recorded contigs related to Endopeptidases, Zinc metallopeptidase, Ubiquitinyl hydrolases, Autocrine proliferation repressor protein A from our tomonts contigs. Although it was not possible to functionally predict/classify these peptidases based on location, this information is valuable for further studies. Similarly, in *C. irritans*, proteases have been identified as therapeutic targets with the application of protease inhibitors [8]. The cysteine protease family, Leishmanolysin domain-containing proteases, from the *C. irritans* transcript database, was predicted and their importance was emphasized as diagnostic markers during infestation by *C. irritans* in mariculture systems and as vaccine candidates based on their localization and the presence of repetitive motifs [23]. In the salmon louse, *Caligus rogercresseyi* serpin-like protein (serine proteases) sequences were differentially expressed during the distinct stages of ontogenetic development [26]. Seven cathepsin-like cysteine peptidases, four serine carboxypeptidases, a eukaryotic aspartyl protease family protein, an ATP-dependent metalloprotease FtsH family protein, three leishmanolysin family proteins and a peptidase family M49 protein were identified from a *Miamiensis avidus* cDNA library by BLAST X search [25]. However, these proteins—being part of AO tomonts—were identified from the non-infective stage, and their presence in the infective stage of the parasite needs to be confirmed prior to further studies on their use in vaccine formulation. Moreover, a quantitative RT-PCR is required to investigate mRNA expression of the identified peptidase genes from AO tomonts in comparison with other stages of AO to establish the most expressed peptidase genes during the life stages of AO.

In this study, we categorized the virulence proteins from AO transcriptome into Adhesin, Invasion, Heat shock protein, Establishment, and Proteases. In addition to data mining for already described virulence factors in related protozoa, a BLAST search using a custom-made virulence database, consisting of the MvirDB [15] and ProtVirDB [16] databases, was performed. Although we identified other virulence genes from the transcriptome of AO tomonts, in this study we described two genes: heat shock proteins 70 and casein kinase. Hsp70 seems to be the most represented group of heat-shock proteins in AO, with transcripts homologous to mitochondrial and cytosolic types identified. It is evident that Hsp70 proteins are highly conserved in all living organisms and they are crucial for the correct folding of stress-accumulated, misfolded proteins preventing protein aggregation [27,28]. In *Trichomonas vaginalis*, their upregulation in response to heat-shock and oxidative stress was reported [29], although RNA sequencing data show their downregulation shortly after the exposure to high oxygen levels [30]. In Grouper (*Epinephelus coioides*), the C-terminal domain of heat shock protein 70 cloned from *C. irritans* (Hsp70C) was tested for its immunostimulatory effects as an adjuvant in combination with Immobilization antigen [31]. Since the protein sequence of Hsp 70 is available, future studies using recombinant proteins should be conducted to determine the biological activity at the protein level in ESB. Moreover, it would be desirable to identify more sequences of virulent proteins from the available AO transcriptome data and to conduct combined research on transcriptional analysis and biological activities of each virulent protein.

## 5. Conclusions

This is the first description of assembled de novo transcriptome of *Amyloodinium ocellatum* tomonts and we established a reference database for subsequent studies. The majority of the contigs had a similarity with *Symbiodinium microadriaticum*. We observed that cellular components (membrane and cell process) and the molecular function (binding) were the major enriched AO transcripts. Potential virulent proteins from AO contigs (HSP70, AP120, Rab11B, Casein kinase II alpha) were identified, and also two virulent proteins (HSP70 and Casein kinase II alpha) from the AO transcriptome database were characterized. Future studies on the gene expression profile of AO dinospores and trophonts stages will facilitate the identification and differentiation of genes involved in all phases of the parasite life cycle and considered as virulence proteins in AO. Moreover, this could provide an insight into the stage-specific functions of AO and regarding the genes involved in the pathogenesis of this disease. Therefore, these data could contribute to the development of AO vaccines and drug targeted treatments.

## Figures and Tables

**Table 1:** Length description of contigs from *Amyloodinium ocellatum* (AO) tomonts.

	Nber	N50 (bp)	Max Length (kb)	Mean Length (bp)	Total Assembly Length (Mb)	GC%
Contigs	175,030	624	5.435	517.77	90.6	56
Contigs mapping against nr	48,188	NA	NA	NA	NA	NA
Contigs assigned to Alveolata	29,691	1191	5.435	875.9	26.6	62

**Table 2:** Taxonomic classification of AO tomonts contigs.

	Assigned	Total No of Contigs	Similarity (%)
Superkingdom	Eukaryota	45,008	93.4
Class	Dinophycaae	25,258	56.12
Species	*Symbiodinium microadriaticum*	23,897	94.61

**Table 3:** Contigs showing the distribution of MEROPS peptidase families.

AO Contig Number	Major Peptidases Family	Content of Family	Functions
Contig_10007	MER1360781-family A31 non-peptidase homologues	Endopeptidases	Cleavage of active enzymes
Contig_22247	MER1200850-family M8 unassigned peptidases	Zinc metallopeptidase	Role in cell migration and invasion
Contig_114405	MER1171318 - family C12 unassigned peptidases	Ubiquitinyl hydrolases	Removal of ubiquitin (Ub) from protein substrates, involvement in numerous biological processes
Contig_32531	MER0253524 - family S82 unassigned homologues	Autocrine proliferation repressor protein A	Regulation of cell proliferation/number

**Table 4:** Contigs showing the distribution of AO potential virulence factors, identified through the custom made virulence protein database.

Virulence Group	AO Potential Virulence Factors	Contig Number
*Adhesin*	AP120	Contig_9557
*Invasion*	Gp63 (surface metalloprotease)	Contig_142760
	P0 (Ribosomal phosphoprotein)	Contig_3026
	ROM1 (Rhomboid-like protease)	Contig_40819
	RabA	Contig_83170
*Heat shock protein*	Hsp70	Contig_50520
*Establishment*	Casein kinase II alpha	Contig_10331
	Rab7A	Contig_103654
	Vps35	Contig_1094
	Rab11B	Contig_112882
	Vps29	Contig_116994
	Rab5	Contig_129899
	CPSII	Contig_60947
*Proteases*	Brucipain	Contig_105984
	Plasmepsin II	Contig_10808
	Plasmepsin I	Contig_12020
	Plasmepsin IV	Contig_19630

## Supplementary Materials

The following are available online at https://www.mdpi.com/2073-4425/11/11/1252/s1, Figure S1: length distribution of AO contigs, Figure S2: Predicted secondary structure of hsp 70 (a) and casein kinase II alpha (b).
