# Supplementary material for: Transcriptome Analysis of Amyloodinium ocellatum Tomonts Revealed Basic Information on the Major Potential Virulence Factors
Source: Genes (Basel). 2020 Oct 24;11(11):1252. doi: 10.3390/genes11111252 (PMC7692099; doi:10.3390/genes11111252)
Supplement: Supplementary file 1 [file genes-11-01252-s001.pdf]

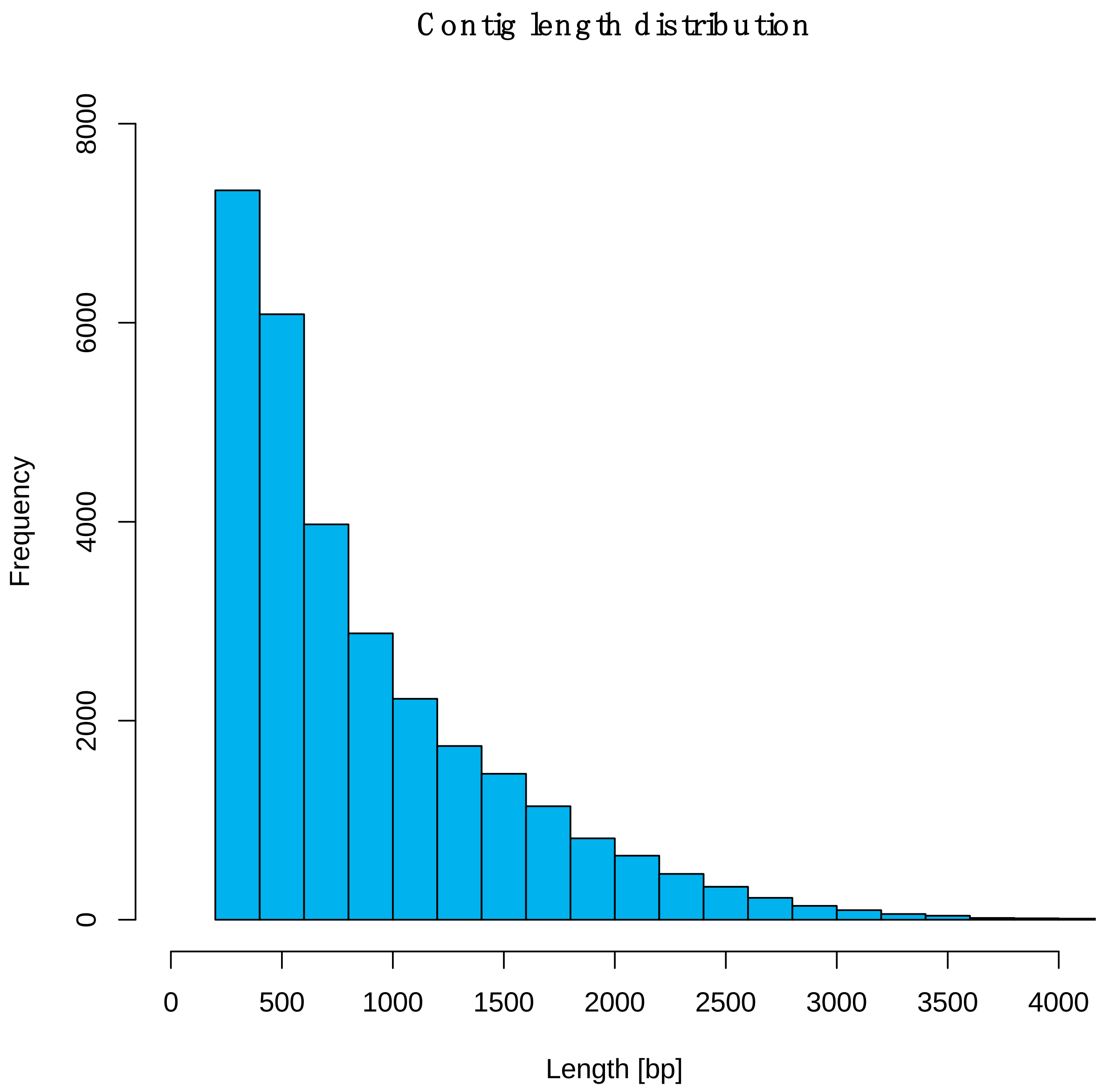

Supplementary Figure 1. Length distribution of assembled AO transcripts.

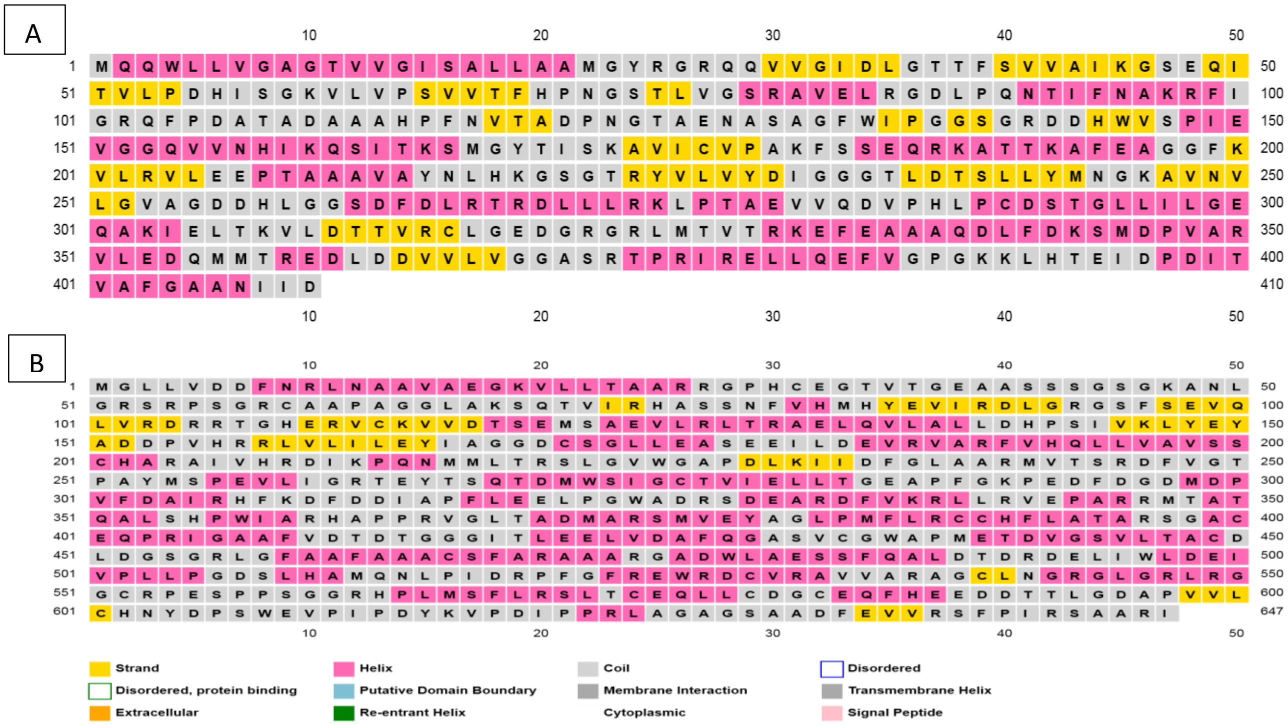

Supplementary Figure 2- Predicted secondary structure of Hsp 70 (a) and casein kinase II alpha (b) protein containing helix as shown.
